# A single session of coordinative motor training does not improve spatial ability performances in healthy children

**DOI:** 10.3389/fspor.2024.1411127

**Published:** 2024-10-14

**Authors:** Christina Morawietz, Anna Maria Wissmann, Till Kuehne, Thomas Muehlbauer

**Affiliations:** Division of Movement and Training Sciences/Biomechanics of Sport, University of Duisburg-Essen, Essen, Germany

**Keywords:** treatment, acute effects, visual-spatial abilities, youth, movement coordination

## Abstract

**Background:**

In recent years, studies have found small-to-medium positive effects of physical activity on academic achievement. Already acute bouts of exercise appear to improve certain cognitive functions. Spatial abilities are one aspect of cognition that is encountered frequently in daily life and that is closely related to success in science, technology, engineering, and mathematics (STEM)-subjects. However, little is known about the effects of an acute exercise session on spatial abilities. The aim of this study was therefore to evaluate the effect of a single session of coordinative motor training (CMT) on spatial ability performances in healthy children.

**Methods:**

Forty-nine children were assigned to either a single session of CMT (i.e., obstacle course with motor coordinative and spatial elements) (*n* = 25, 12 females, mean age: 10.7 ± 0.6 years) or a resting control group (*n* = 24, 12 females, mean age ± SD: 11.4 ± 0.5 years). Spatial abilities were evaluated in both groups using the Paper Folding Test (PFT), Mental Rotation Test (MRT), Water Level Task (WLT), Corsi Block Test (CBT), and Numbered Cones Run (NCR).

**Results:**

A statistical main effect for Test was observed for the majority of outcomes (i.e., all but the MRT). Test × Group interactions did not reach the level of significance.

**Conclusion:**

The results indicate that a single session of CMT does not improve spatial ability performances of healthy children. Future research should evaluate whether repeated longer-term interventions might be more suitable to generate significant improvements in spatial abilities.

## 1 Introduction

The positive effects of physical activity and exercise on physical and mental health as well as quality of life are recognized widely (1–3). Not only does the risk for cardiovascular disease, diabetes, obesity, and musculoskeletal disorders decrease with increased physical activity, there also appear to be benefits for cognitive functions and brain health (3–5).

In recent years, a variety of studies have investigated the connections between physical activity and aspects of academic achievements in school aged children and adolescents indicating a small-to-medium positive relation between the two factors (e.g., 6–8). Considering the deteriorating results of the recent OECD PISA-study 2022 in combination with global health problems like overweight and noncommunicable disease, this positive association could be a way to address several of today's problems at once (9, 10).

Spatial abilities are one aspect of complex cognitive function that is not only encountered frequently in daily life (e.g., wayfinding, problem solving or map reading) (11, 12) but is also closely linked to academic achievements, particularly to success in STEM-subjects (i.e., science, technology, engineering, mathematics) (13–15). Spatial abilities should therefore receive particular attention in the school curriculum (16, 17).

Starting to develop from early infancy on, maturation of spatial abilities continues right into adolescence and appears to be closely linked to the ongoing development of motor abilities, sensory systems, and brain maturation (18, 19). Increasing motor independence allows infants new ways to explore and interact with their environments which aids spatial development (e.g., spatial knowledge and perception) (19, 20). The development of spatial orientation in particular has been linked to the maturation of hippocampal and parahippocampal areas. Moreover, the frontal (i.e., dorsolateral prefrontal, medial and superior frontal lobe), temporal (i.e., inferior, superior and medial temporal lobe), parietal (i.e., superior and inferior parietal lobe) and occipital (i.e., precuneus) lobe, cingulate (i.e., retrosplenial cortex) and motor (i.e., premotor cortex) cortices as well as subcortical structures (i.e., thalamus, striatum, cerebellum) appear to be relevant for spatial orientation (12, 18, 21–23). While there is a large overlap in brain activation patterns of children and adults regarding spatial orientation and navigation abilities, children and adolescents show increased central nervous system (CNS) activity (e.g., in brain areas related to visuospatial functioning) during navigational activities compared to adults (18, 24, 25). This might indicate that CNS structures are still maturing and neural connectivity as well as efficient use and switch between orientation strategies (e.g., allocentric or egocentric) still need to be acquired or enhanced (18, 24).

While it has been shown that spatial abilities can be trained using paper-and-pencil and computer-based tasks (17, 26) recent research also found that coordinative motor training (CMT) approaches lasting for several weeks positively impact on children's spatial abilities (27–30). Jansen and Richter (31) further detected that a single session of creative dance training (i.e., training of orientation in space) resulted in superior mental rotation (i.e., one aspect of spatial abilities) than regular PE-class in second graders (*n* = 64; mean age ± SD: 7.09 ± 0.73 years).

In line with these findings, a positive impact of single exercise sessions on cognitive functions and based on this also on academic achievements have been reported by several researchers (e.g., 31–35). The adaptive mechanisms underlying theses positive effects, however, are not entirely understood to date and studies examining the neurophysiological backgrounds are limited and vary considerably in methodology (36). Research in children suggests that acute bouts of exercise may cause functional adaptations in the CNS like prompt neurochemical responses improving cognition (e.g., growth factors like brain derived neurotrophic factors) or different functional brain activation patterns (for reviews see 2, 37, 38). Regarding training content, Voelcker-Rehage and Niemann (39) and Meijer et al. (40) point out that there is a significant research gap when it comes to investigating the effect of CMT (acute and chronic) on CNS adaptations (functional and structural) and cognitive functions with the majority of research implementing cardiovascular interventions.

An exemption is the study by Mochizuki and Kirino (41) evaluating the neural activation patterns (fMRI) during a single session of watching or performing coordination exercises compared to control exercises. Results indicate a noticeable overlap between CNS structures addressed with these coordination exercises and those required for the conduction of spatial orientation or navigation tasks. These findings are supported by studies performing longer term CMT (e.g., 42, 43). Particularly, parietal and occipital areas, the prefrontal cortex, premotor areas, and subcortical structures seem to be involved in spatial and coordinative activities (39, 41–43). When evaluating the effect of acute CMT on attention and concentration, Budde et al. (44) further suggest that CMT is associated with higher cerebellar and frontal lobe activation, structures also involved in spatial orientation.

With many children spending a majority of their time in school or childcare facilities, these environments are essential to provide cognitively stimulating surroundings and promote physical activity as well as academic success (45). However, time is limited, school curricula have little flexibility and many subjects and socioeconomic topics require consideration (46). It is therefore of great interest to pursue research on the effect of physical activity interventions (e.g., CMT) that are little time-consuming (i.e., single bouts of exercise) on specific cognitive functions like spatial abilities and academic success.

The purpose of this brief research report is to evaluate the effect of a single session of CMT (i.e., obstacle course with motor coordinative and spatial elements) on spatial abilities of healthy children. Previous research has already shown positive effects of a longer-term coordinative motor intervention on spatial abilities using the same measurement tools in the same population (30). Building on this, in a next step we planned to evaluate the effects of an acute intervention. Initial evidence by Jansen and Richter (31) has shown beneficial effects of an acute motor training intervention on mental rotation (i.e., one aspect of spatial abilities) in healthy children. Moreover, previous research finds overlapping brain activation patterns regarding spatial orientation and CMT indicating that activation of these areas during CMT could also improve spatial functions by e.g., aiding functional connectivity, increasing cerebral blood flow or neurochemical adaptations (40–44, 47). Based on these findings and aiming to contribute to this still relatively limited field of research, we hypothesized that participation in a single session (i.e., 30 min) of CMT would result in superior spatial ability performance compared to a resting control group (i.e., no training at all) as measured by five established spatial ability tests. Brief active movement breaks during cognitively challenging subjects and breaks between classes that encourage movement are already used within the school context to facilitate concentration and attention, improve posture and promote general motor skills (48, 49). They are, however, not entirely established yet and can be subject to objections and fear of loss of control in educational staff (49). Structured approaches like this acute CMT might have the potential to generate additional effects on specific cognitive functions like spatial abilities and thereby contribute not only to health, wellbeing and better learning conditions but also address academic achievement and improve the currently limited base of empirical evidence on this topic.

## 2 Methods

### 2.1 Participants

Using G*Power (version 3.1.9.7) the power analysis (*f* = 0.29, *α* = 0.05, 1−*β* = 0.80, number of groups: *n* = 2, number of measurements: *n* = 2, correlation between testing: *r* = 0.40, drop-out rate per group: 10% due to reasons not attributable to treatments) was calculated from a previous study on acute effects of training on spatial ability in children (31) and revealed that a total sample size of *N* = 36 participants would be sufficient to detect medium-sized treatment effects (50). A total of forty-nine healthy children participated in this study and were randomly assigned to an intervention group (*n* = 25, 12 females, 13 males, mean age ± SD: 10.7 ± 0.6 years) or a control group (*n* = 24, 12 females, 12 males, mean age ± SD: 11.4 ± 0.5 years). All participants attended the fifth grade of public secondary schools in the Ruhr area of North Rhine-Westphalia, Germany. The training intervention and assessments were unknown to the participants. All participants and their legal guardians provided informed consent before entering the study. The study protocol was approved by the Human Ethics Committee of the University of Duisburg-Essen, Faculty of Educational Sciences (EA-PSY20/23/04102023).

### 2.2 Experimental design

**Figure 1:**
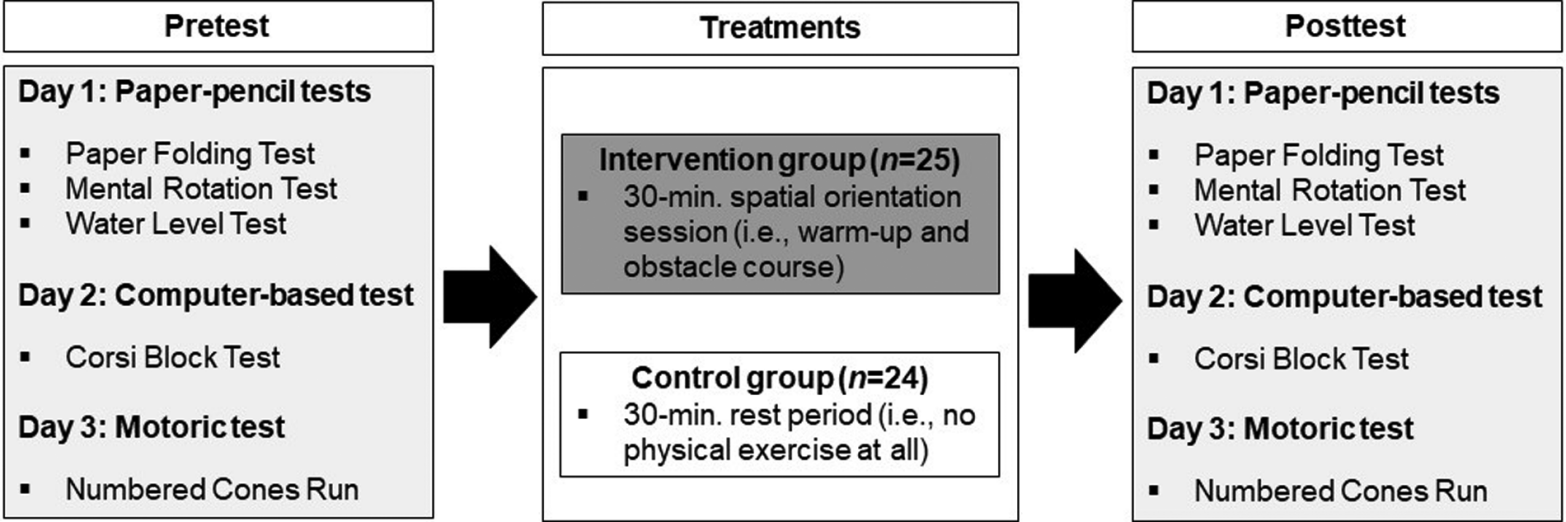
Schematic description of the study design.

The study was conducted within a school setting. Group allocation was performed by drawing lots and based on class affiliation. The class assigned to be the intervention group (INT) performed a single 30-minute CMT, while the class assigned to be the resting control group (CON) performed no training at all (Figure 1). Before and after the intervention or resting a spatial ability assessment consisting of five established spatial ability tests was conducted with the participants from both groups. The assessment procedure (i.e., pretest and posttest) was spread over three testing days and took place in the mornings. On the first day, three paper-and-pencil assessments were performed with the entire class in the classroom. A computer-based test was conducted on the second day. It was instructed in small groups of five participants and then carried out individually in a separate room. Lastly, on the third day, a motoric test was instructed and conducted in small groups of five participants in the school gym. A graduated sports scientist conducted the testing procedures. The single training session was implemented by a second graduated sport scientist.

### 2.3 Measures

**Figure 2:**
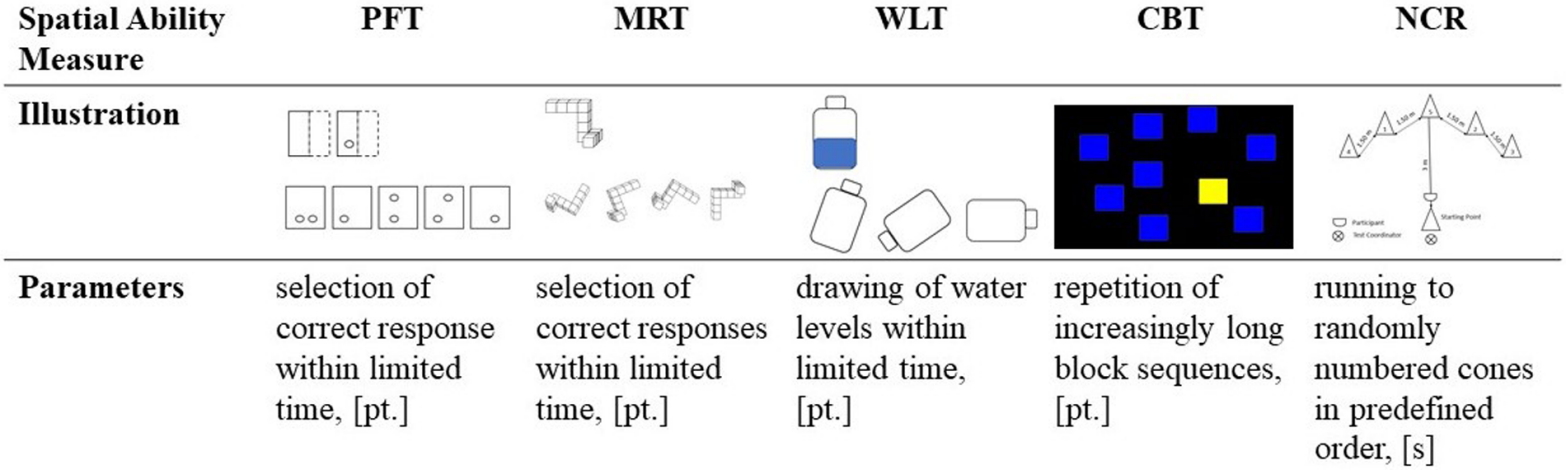
Schematic depiction of the spatial ability measures.

The spatial ability assessment took place on three testing days and included three paper-and-pencil measures (i.e., Paper Folding Test [PFT], Mental Rotation Test [MRT] and Water Level Task [WLT]), one computer-based measure [i.e., Corsi Block Test (CBT)], and one motoric test [i.e., Numbered Cones Run (NCR)]. The testing procedure was identical to the one used in our previous work (30). A brief depiction of the measures applied can be found in Figure 2. All measures were proven to be reliable for the age group presented in this study [i.e., PFT: intraclass correlation coefficient (ICC) = 0.78, MRT: ICC = 0.81, WLT: ICC = 0.88, CBT span: ICC = 0.95, CBT composite score: ICC = 0.93, NCR: ICC = 0.91] (51).

### 2.4 Intervention

**Figure 3:**
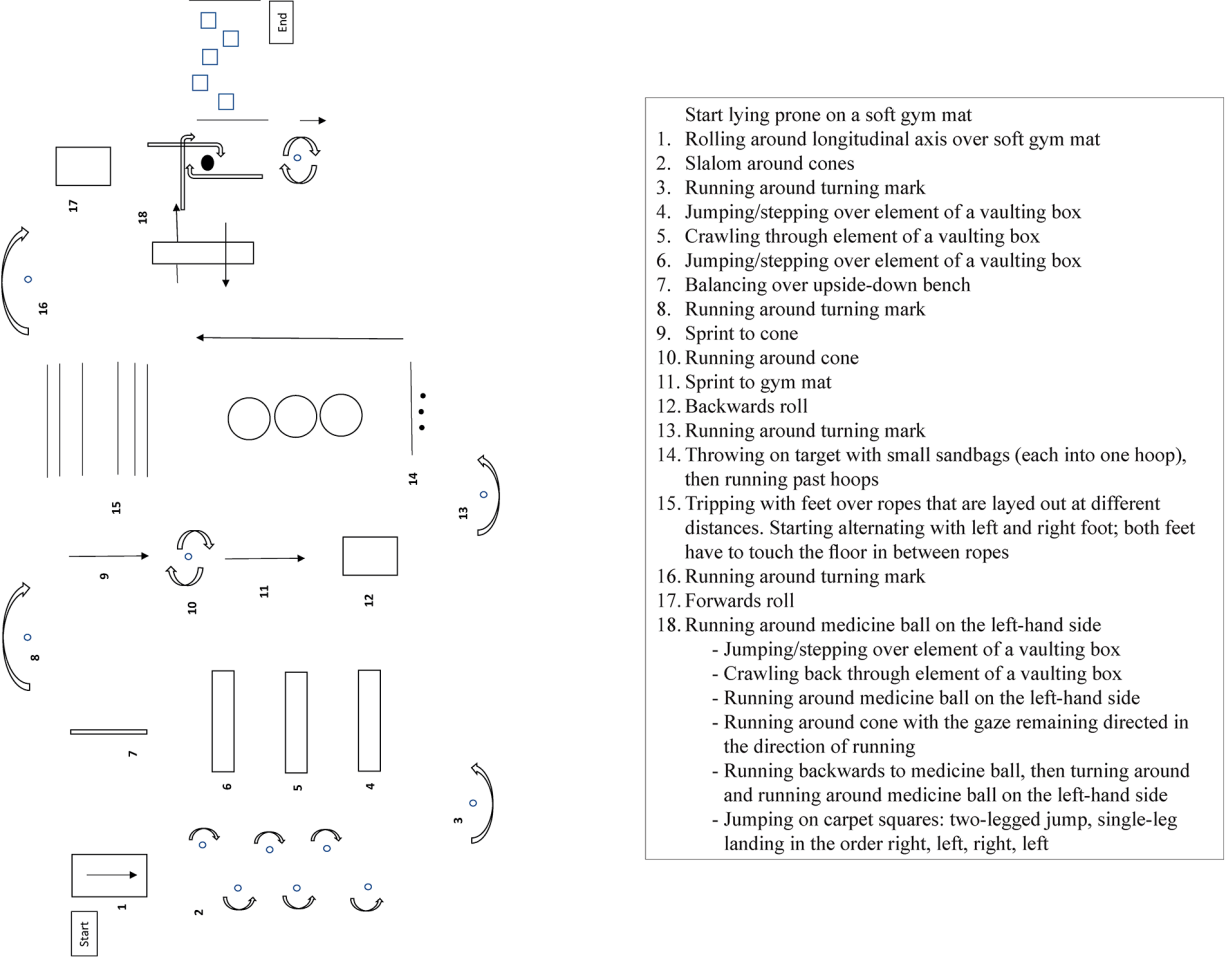
Schematic depiction of the intervention.

The intervention was conducted during regular PE-class and consisted of brief warm-up (5–10 min) followed by a main part that was conducted for approximately 20 min. During the warm-up period, students first had to find the best path through eight hoops that were distributed on a square of 10 m × 10 m. Then, paths of fellow students had to be replicated. There were four different starting points. The warm-up was based on suggestions for skill-oriented training of spatial orientation by Hirtz et al. (52) as well as the LifeKinetik® program by Lutz (53). The main CMT consisted of an obstacle course that had to be completed ten to twelve times by each participant in different running directions (i.e., running forward, backward, and sideways) (Figure 3). Participants were asked to focus on movement accuracy rather than speed. Compliance was visually monitored and participants were verbally motivated by the graduated sports scientist conducting the intervention. The obstacle course consisted of coordination exercises with spatial elements that were derived from various tests for motor abilities and coordination (e.g., Hamburg Parcours, Koko-Test, Swiss Cross, Mini-Biathlon) (52, 54–56). Tasks included amongst others rolling sideways, forwards, and backwards, slalom running, climbing over, and passing under obstacles, balancing, running with changes of direction, target throwing, jumping, rotations, and single leg landing.

### 2.5 Statistical analysis

Data were presented as group mean value ± SD. For all analyses, assumptions of normality (Shapiro–Wilk Test) and homogeneity of variance/sphericity (Mauchly Test) were checked and met prior to conducting parametric analyses. Afterwards, performance differences between groups at the pretest were checked and detected for the WLT, CBT CS, and NCR. Consequently, a series of baseline-adjusted 2 (test: pretest, posttest) × 2 (group: INT, CON) repeated measures ANOVA were performed. Where significant Test × Group interactions were detected, *post hoc* analyses (i.e., paired *t*-test) using Bonferroni-adjusted *α* determined the location of any differences. The significance level was *a priori* set at *α* < 0.05. For the ANOVA, effect size was calculated as partial eta-squared (*η*_p_^2^) and reported as small (0.02 ≤ *η*_p_^2^ ≤ 0.12), medium (0.13 ≤ *η*_p_^2^ ≤ 0.25), or large (*η*_p_^2^ ≥ 0.26) (57). For the paired *t*-test, effect size was calculated as Cohen's *d* and stated as small (0 ≤ *d* < 0.50), medium (0.50 ≤ *d* < 0.80), or large (*d* ≥ 0.80) (57). All analyses were performed using SPSS version 28.0 (IBM Inc., Chicago, IL).

## 3 Results

Table 1 displays descriptive and inference statistics for all analysed variables. Baseline differences between groups were observed for the WLT, the CBT CS, and the NCR. The baseline-adjusted statistical analysis showed significant main effects of Test for all but one outcome (i.e., MRT: *p* = 0.095), indicating improvements from the pretest to the posttest, irrespective of group. However, the Test × Group interactions did not reach the level of significance (i.e., PFT: *p* = 0.492; MRT: *p* = 0.051; WLT: *p* = 0.622; CBT span: *p* = 0.497; CBT CS: *p* = 0.341; NCR: *p* = 0.688). This indicates that no group-specific changes occurred from the pretest to the posttest. Additionally performed gender-specific sub-analysis did not reveal any significant Test × Group interactions (data not shown).

**Table 1:** Acute effects of a single coordinative motor training session on measures of spatial ability performance in healthy children by group.

Outcome	Intervention group (*n* = 25)		Control group (*n* = 24)		*p*-value (*η*_p_^2^)	
	Pretest	Posttest	Pretest	Posttest	Main effect: Test	Interaction effect: Test × Group
Paper Folding Test [pt.]	8.0 ± 3.3	9.5 ± 3.0	7.0 ± 3.3	9.3 ± 3.5	<0.001 (0.25)	0.492 (0.01)
Mental Rotation Test [pt.]	6.1 ± 2.7	10.4 ± 4.9	7.0 ± 3.4	9.0 ± 5.6	0.095 (0.06)	0.051 (0.08)
Water Level Task [pt.]	7.4 ± 2.3	8.0 ± 2.3	5.1 ± 3.5	6.5 ± 3.4	<0.001 (0.27)	0.622 (0.01)
Corsi Block Test span [pt.]	4.9 ± 0.9	5.4 ± 1.2	5.3 ± 1.3	5.4 ± 1.2	0.002 (0.19)	0.497 (0.01)
Corsi Block Test CS [pt.]	31.5 ± 12.4	41.2 ± 18.1	41.1 ± 17.5	43.6 ± 19.8	0.007 (0.15)	0.341 (0.02)
Numbered Cones Run [s]	9.1 ± 1.5	8.9 ± 1.1	10.5 ± 1.3	9.9 ± 1.2	<0.001 (0.26)	0.688 (0.01)

Values are means ± standard deviations. Threshold values for the *η*_p_^2^-value were 0.02 ≤ *η*_p_^2^ ≤ 0.12 = small, 0.13 ≤ *η*_p_^2^ ≤ 0.25 = medium, and *η*_p_^2^ ≥ 0.26 = large. CS, composite score.

## 4 Discussion

The aim of this brief research report was to investigate the effect of a single session of CMT on spatial ability performances of healthy children. It was hypothesized that participation in the CMT would have superior effects on spatial abilities than no training at all as measured by five established spatial ability tests. The results, however, do not support our hypothesis. No significant Test × Group interactions could be observed in the present study, indicating that changes in spatial ability performance from pretest to posttest cannot be attributed to either INT or CON.

Overall, many studies evaluate the effect of acute bouts of exercise on cognition in children or adolescents. However, many of those focus on executive functions with some others analyzing cognitive functions like e.g., attention, working memory or information processing (e.g., 33, 58–61). To our knowledge, though, only one study investigates the effect of an acute bout of exercise (i.e., creative dance training) on spatial abilities (i.e., mental rotation) in children (31). Comparable studies in different populations are also scarce. Only one study evaluating the effect of either 45 min of physical activity (i.e., running, jumping, rope skipping, and callisthenic) or a lecture on kinematics on mental rotation performance in healthy young adults could be identified (62). Comparison of our findings to other research in the field is therefore limited. While both studies mentioned above reveal that the exercise interventions resulted in superior mental rotation performance than the control conditions, mental rotation was only one of the spatial abilities tested in the present research. Moreover, participant age, differences in testing procedures and tools used to evaluate mental rotation as well as intervention content and duration [i.e., 60 min (31) and 45 min (62) respectively vs. 30 min (present study)] need to be taken into consideration. Even though both studies can be considered an indication that acute bouts of exercise might influence mental rotation, it is impossible to predict the impact of acute bouts of exercise on other spatial abilities. More high-quality studies are needed in this research field that focus on dose-response relationships in association with participant age and the type of cognitive function measured.

The CMT performed in the present study might further not have been specific or challenging enough to generate significant improvements in spatial abilities. Generally, cognitively more challenging types of exercise or open skill exercises (OSE) appear to yield superior results to closed skill exercises (CSE), however, research regarding the role of exercise type on cognitive abilities is still inconclusive (2, 58, 63). OSE are characterized by unpredictable or dynamic environments and have higher cognitive demands as they require extrinsic abilities, decision making, and reaction to present situations, whereas CSE can be identified as motor tasks performed in stable and predictable environments that are cognitively less demanding (64, 65). Environmental requirements in the present study were rather predictable for the participants, particularly since they completed the course ten to twelve times each. It can therefore be considered a CSE. OSE focusing on coordination might thus have been more efficient in improving spatial abilities. At the same time O’Brien et al. (60) reported that a single session of OSE resulted in improved verbal working memory and a single session of CSE improved motor working memory. However, neither benefitted visuospatial working memory as measured by a computerized CBT, which is one of the measurement tools also used in the present study.

Even though CMT parameters might be more complex to regulate than other motor skills (e.g., strength, endurance, flexibility), it is indispensable to establish a monitoring system to control intervention parameters. In the present study, participants’ performance on the CMT was controlled in a quantitative as well as qualitative way. Quantitative control was based on the following parameters: training duration (i.e., 20 min), frequency (i.e., 10–12 rounds/participant), duration per round (i.e., 90–120 s), intensity (i.e., moderate speed), and rest between rounds (i.e., 0–30 s). Qualitative control was executed by the graduated sports scientist conducting the CMT. Participants’ compliance was visually monitored and maintenance of performance was encouraged verbally.

Still, exercise intensity might be a determining factor for spatial ability outcomes. Even though, participants were asked to complete the obstacle course rather accurate than fast, no precise statement can be made about exercise intensity, as we did not control participants’ heart rate (HR). Neither Jansen and Richter (31) nor Jansen and Pietsch (62) provide detailed information on exercise intensity for their creative dance and their physical activity interventions respectively. Research evaluating the effect of acute bouts of exercise on other cognitive functions are not conclusive in terms of exercise intensity and outcomes (4). Hillman et al. (33) found positive effects on cognitive control and attention after 20 min treadmill walking at an estimated 60% of the maximum HR in healthy children. Similarly, positive effects on some executive functions were found by Chen et al. (34) after either jogging for 30 min at moderate intensity (60%–70% of maximum HR) or reading. Budde et al. (44) discuss superior effects on a concentration and attention task when comparing coordinative exercises with a normal sports lesson in adolescents performed at approximately 60% of the maximum HR. van den Berg et al. (66) on the other hand reported that adolescents (mean age ± SD: 11.7 ± 0.7 years) performing either 12 min of aerobic, coordination or strength exercises at comparable or slightly lower intensities did not improve information processing and selective attention. Also Bedard et al. (67) and Best (68) were not able to determine superior outcomes of exergaming interventions at 75% and 70%–80% of the maximal HR respectively over control conditions on executive functions in children (mean age ± SD: 7.04 ± 1.37 years and 8.1 ± 1.3 years respectively). More research is needed to evaluate the optimal dose-response relationship with regard to the participants’ age. Moreover future research needs to take a closer look at different training intensities and the particular cognitive functions targeted at theses intensities (69).

The results further showed a main effect of Test for the majority of outcomes measured (i.e., all but the MRT), indicating that performance on those spatial ability measures improved significantly from pretest to posttest for children in the INT and in the CON. As there appears to be no superiority of the intervention, findings might be suggestive of retest effects. Retest effects are common in cognitive testing and stand for improvements in test results when repeatedly performing the same test (70, 71). While retest effects can be reduced by longer test-retest intervals, they cannot be prevented completely and therefore need to be taken into account when interpreting study results as otherwise findings are easily overestimated (70). Previous studies have already reported retest effects on some of the measures used in this study (i.e., PFT and CBT) (72, 73). Even though retest effects also occurred in one of our previous studies, we were still able to demonstrate that all spatial ability measures applied in the present study are reliable and can be used to detect intervention changes in the represented age- and population group (51). Based on the results, one can therefore assume that the acute CMT intervention did not or only marginally impact on the participant's spatial ability performance.

### 4.1 Limitations and directions for future research

Several limitations of the present research need to be taken into consideration for future research. Only healthy children participated in this study. Results can therefore neither be generalized to other age groups or populations nor to other types of exercise or assessments.

Although the present study was adequately powered (i.e., 80%) to investigate its hypothesis, the relatively small sample does limit the generalisability of the findings. Studies that replicate and extend our findings could increase the statistical power and thus the probability of detecting an effect by increasing the sample size.

To date there is no standardized way to evaluate the effect of acute bouts of exercise on cognitive and particularly spatial abilities and studies vary widely in methodology. Controlling parameters like HR, ratings of perceived exertion or step counts could help to gain deeper insights into exercise intensities. Other moderating variables like dosage, timing of the testing procedure and the type of cognitive demand tested also need to be taken into consideration when conducting future research (32, 69, 74, 75).

Even though the suitability of the applied spatial ability measures for this age group was established previously (51), tests might not have been specific enough to capture improvements in spatial abilities resulting from the CMT. For future research, more appropriate standardized, established and accessible measures might be determined that allow reproducible evaluation of spatial abilities after acute motor training interventions.

Moreover, little is known about the sustainability of acute exercise effects on cognitive functions and specifically spatial abilities. Most studies evaluate the effects immediately after the intervention and only few researchers conduct a follow-up to establish how long potential immediate effects last (76). Future research should take follow-up assessments into consideration to identify the efficiency and durability of acute exercise effects on cognitive functions.

## 5 Conclusion

In conclusion, the hypothesis that a single session of CMT improves spatial ability performances of healthy children could not be confirmed. To date, little is known about the optimal intervention content dosage, timing of testing, cognitive demands tested, measurement tools or sustainability in relation to single bouts of exercise and cognitive functions. Future research should focus on these topics as findings may be of great value to tackle sedentary behavior and at the same time foster complex cognitive functions like spatial abilities. The rejection of our hypothesis suggests that repeated longer-term interventions might be more suitable to generate significant improvements in spatial abilities, which implies an additional field of research.

## Data Availability

The raw data supporting the conclusions of this article will be made available by the authors, without undue reservation.
